# Protocol Paper: Conducting Life History Interviews to Explore the Journeys of People with Disability from Syrian and Iraqi Refugee Backgrounds Settling in Australia

**DOI:** 10.3390/ijerph18157978

**Published:** 2021-07-28

**Authors:** Angela Dew

**Affiliations:** School of Health and Social Development, Institute of Health Transformation, Deakin University, Geelong 3211, Australia; angela.dew@deakin.edu.au; Tel.: +61-3-9244-5766

**Keywords:** refugee, disability, journeys, participatory action research, life history interviews, Syria, Iraq, Australia

## Abstract

This paper outlines a research protocol to be undertaken with people with disability from Syrian and Iraqi refugee backgrounds settling in Australia. Since 2012, the numbers of people with disability arriving from these countries has increased with limited understanding about the impact of their refugee journeys on their settlement. The aim of this small-scale exploratory study is to learn about the journeys made by people with disability from Syrian and Iraqi refugee backgrounds from their countries of origin, through transit countries, to Australia in order to understand the impact of these journeys on inclusion and participation in Australian society. This participatory action research study employs a bilingual co-researcher with disability from a Syrian background to conduct life history interviews with up to five participants. Participants will recount their journeys with a focus on the impact of their disability on this experience. The study design is informed by BenEzer and Zetter’s 2014 seminal paper on the importance of the refugee journey to settlement. This study has the potential to foreground the voices and experiences of people with disability from refugee backgrounds who are often absent, silenced or excluded in research and, in so doing, hopefully impact Australian refugee policy.

## 1. Introduction

There are an estimated 10 million displaced persons with disability globally [1]. The disability-related needs of these people from diverse ethnic, cultural, linguistic and religious backgrounds are often ignored, leading to increased marginalisation, disadvantage and exclusion [1]. In 2008 Australia ratified the United Nations Convention on the Rights of Persons with Disabilities (CRPD) [2], with a subsequent shift in public policy towards increased resettlement of refugees with disability [3,4]. Importantly for this group, in 2012, the Australian Government streamlined the health waiver for humanitarian visa applicants so that the costs of health or community care services were no longer assessed [5]. The result of this waiver is that people with disabilities are increasingly applying for, and being granted, offshore humanitarian refugee visas [6,7]. An estimated 1.4% of the 17,555 Australian humanitarian visa recipients in 2015–2016 received a health waiver indicating disability or chronic illness [8]. A 2019 Refugee Council of Australia report highlighted that very little is known about the impact of disability on the resettlement experiences of refugees or the understanding and resources of local communities to facilitate successful settlement [8]. As evidenced by the divisive public discourse and political response, there is seemingly limited understanding among many Australians about the potentially long-lasting effects of the perilous and often traumatic journeys people make to reach a country of refuge and the impact of this on their physical and emotional health and wellbeing. This lack of understanding may be particularly marked for those who seek refuge with a disability, as they have often experienced a lifetime of discrimination and marginalisation [1].

Article 1 of the CRPD defines disability as “…long term physical, mental, intellectual or sensory impairments, which in interaction with various barriers may hinder [the person’s] full and effective participation in society on an equal basis with others” [2]. The CRPD highlighted the importance of environmental barriers and social contexts to the experience of disability [2], including in Article 11 for those at risk due to war and humanitarian emergencies [3]. As a signatory to both the CRPD and the 1951 Refugee Convention [9], Australians need to understand and respond to the factors that impact a person with disability from a refugee background’s resettlement throughout their journey from country of origin, through transit countries, to Australia [10].

In a review of the published and grey literature, Soldatic et al. [11] (p. 502) noted in Australia, the “invisibility of refugees and asylum seekers with disabilities in both disability literature and refugee settlement research”. As indicated by this review, there is currently limited evidence about and understanding of, from the viewpoint of those with lived experience, the often traumatic journeys of refugees pre-arrival, and the ongoing trauma experienced by some post-arrival [10]. Soldatic and colleagues [11] commented that the lack of data collected on people with disability from refugee backgrounds in Australia reinforces their invisibility within the community. As noted previously, an understanding of people’s experiences is essential to ensuring successful resettlement for people with disability from refugee backgrounds [12].

In their widely cited paper on refugee journeys, BenEzer and Zetter [13] (p. 297) proposed that while refugee journeys are “profoundly formative and transformative” (p. 301) experiences they are often overlooked in research with focus rather on the leaving and the arriving meaning the “exilic process” (p. 298) is forgotten. Drawing on BenEzer and Zetter’s work, Crawley and Jones [14], explored the experiences of Syrian, Nigerian and Afghani refugees and noted that refugees are “active agents in formulating their own plans albeit within often constrained opportunities” (p. 3228). These authors described, despite the varied countries of origin and exilic experiences of their participants, the importance to refugees of a settled family life, the ability to earn money and access to housing, education and healthcare. Crawley and Jones [14] concluded that the refugee journey is “embedded within a larger story arc of events and experiences which determine how they travel, where they go and why” (p. 3234). BenEzer and Zetter [13] contest that long after resettling in a new country, the experience of the refugee journey influences people’s recollections of their pasts and how they construct their present lives. They describe the journey as “a powerful notion in the human psyche” (p. 301) with positive (e.g., courage, resourcefulness and personal growth) as well as negative (e.g., trauma, loss and persecution) impacts. BenEzer and Zetter [13] described four conceptual challenges in understanding refugee journeys: temporal characteristics (when the journey is considered to start and end); drivers and destinations (reasons and motivations for the journey); the process/content of the journey (what happened during); and the characteristics of the wayfarers (individual and group demographic characteristics). Notably, despite the high rates of disability among people from refugee backgrounds, disability is not identified by BenEzer and Zetter as a wayfarer characteristic [13].

## 2. Aim

Building on previous work conducted by the author and colleagues [15,16], and applying BenEzer and Zetter’s [13] conceptual challenges to this previously unrecognized group, the aim of this study is to learn about the journeys made by people with disability from Syrian and Iraqi refugee backgrounds from their countries of origin, through transit countries, to Australia in order understand the impact of this “formative and transformative” experience [13] (p. 297) and better support people’s inclusion and participation in Australian society. The aim of publishing this prospective study protocol is to provide a guide for others considering participatory action and life history research involving marginalized populations, including those from culturally and linguistically diverse backgrounds.

## 3. Materials and Methods

The study commenced in February 2021, data collection is scheduled to commence in July 2021, and the study will be completed in January 2022. At the time of writing this paper, a co-researcher has been appointed and ethical clearance for the study received from Deakin University Human Research Ethics Committee.

### 3.1. Lived Experience Engagement Strategy

Reflective of the disability rights movement dictum “nothing about us without us” [17], this study uses a participatory action research (PAR) methodology. An Arabic-speaking person with disability who came to Australia from Syria has been employed as a co-researcher to conduct life history interviews alongside the author. PAR is a collaborative methodology with agreement on goals, data gathering and analysis, and implementation and dissemination strategies with the aim of raising consciousness, addressing crucial needs, and promoting change in the lives of those involved [18]. The use of a PAR approach will (i) produce knowledge and action that is directly useful to individuals and organisations and (ii) increase community awareness of complex needs, problems, attitudes or behaviour [18], and in the case of this study, the journeys made by people with disability from Syrian and Iraqi refugee backgrounds. Information will be gathered using life history interviews.

Life history interviews are a qualitative data collection method, with foundations in oral history, where participants describe in-depth life experiences related to a specific topic over a period of time [19,20,21]. Through these personal accounts, participants explore and identify their dominant narratives—for this study, narratives related to their disability and refugee journey [13]. Life histories enable participants to recollect the past and document change. As described by BenEzer and Zetter and Crawley and Jones, the temporal nature of participants’ refugee journeys will be important to explore in this study [13,14]. As a participatory method, life histories amplify participants’ voices and equalise the inherent power imbalance between researcher and participant. Participants have a prominent role in deciding what to talk about, why it is significant, and to position themselves within the experience [21]. This approach situates participants’ personal experiences within a changing social context which is particularly appropriate for understanding refugee journeys.

The author and co-researcher are working collaboratively to develop their confidence and skills in conducting life-history interviews. This preparation includes discussing how to ask questions sensitively and respectfully to gather information about the journeys made by refugees with disability. Davies et al.’s [19] guide to conducting life history interviews is a useful practical tool in jointly preparing the author and co-researcher for the interviews. The guide advocates a before, during and after interview process to ensure researchers are well prepared and responsive. Bicultural research involving a bilingual co-researcher is preferable to a monolingual researcher working with an interpreter [20]. This is because the bi-lingual co-researcher brings not only a familiarity of language and culture [19] but also contributes their understanding of the research aims and is invested in the research outcomes. In this way, the bilingual co-researcher is able to “convey the underlying cultural meaning of the participant’s words and expressions” [20] (p. 138).

### 3.2. Sample and Recruitment

This is a small-scale, in-depth, exploratory study with a limited 12-month timeframe. Up to five participants will be recruited using purposive sampling [22] via researcher contacts with key refugee support and advocacy organisations. Purposive sampling is used to identify and include participants with lived experience and deep knowledge of the study focus. As described by Patton [22] purposive sampling involves “selecting information-rich cases for study in depth” (p.264). The quality of the information gained is more important than the number of participants. The in-depth nature of life history interviews will generate a large amount of rich data, meaning the small number of participants will provide sufficient preliminary information about the topic [23].

Participant inclusion criteria is as follows: adult (18 years+), living in Melbourne, Australia, with physical, sensory, intellectual/cognitive disability acquired prior to leaving country of origin, of Iraqi or Syrian refugee background with the ability to communicate verbally in Arabic and/or English or using an augmentative and alternative communication system or sign language. Exclusion criteria include the following: disability acquired in transit or since arriving in Australia. The reason for this exclusion is because the study focuses on understanding how disability impacts on participants’ refugee journeys to Australia.

Intellectual or cognitive disability may impact on participants’ ability to provide informed consent and to take part in interviews. Potential participants identifying with intellectual or cognitive disability will be encouraged to seek the support of a trusted person when making the decision about participation and, if desired, have them present during the interviews. If a participant has a formal guardian/support relationship, the consent form will be co-signed by that person/organisation. The researchers will be guided by the person themselves as to whether they require and want additional support for either the consent process or interviews. This support will help to ensure the person understands the consent process and interview questions. At the time of consent, all potential participants will be required to adequately understand and explain the research process to the researcher. This will involve the co-researcher providing a verbal explanation of what is required for participation as outlined in the plain language statement and consent form. The person will then be asked three straightforward questions from the plain language statement to confirm that they understand what participation means. Any participants who cannot adequately explain the research purpose and their participation role will not be included in the study.

## 4. Data Collection

Three two-hour interviews will be conducted with each participant in either Arabic and/or English and audio-recorded with participants’ permission for transcription and translation. In recognition of the time invested, each participant will be given a $50 gift voucher per interview. Each interview will cover one of the identified journey contexts, with one interview focussed on resettlement in Australia, one on transit country experiences, and one on participants’ experiences in their country of origin. The reverse chronological order of the interviews is deliberate to provide participants with an opportunity to become comfortable with the interview process and interviewers by describing their current situation before discussing potentially more traumatic material such as fleeing war and persecution in country of origin and transit [24].

Life history interviews are by their nature largely unstructured to allow participants the opportunity to discuss what is most important to them, and to take the time they need to do so [24]. Jessee [24] describes life history interviews as “a co-creation between the interviewee and the interviewer” (p. 426) designed to “[reveal] the multiple truths of people’s lived experiences” (p. 248). Interviewers must engage in active listening so they can respond to information previously revealed by participants and ask questions to reveal new information about the topic. They must also be responsive to body language and other non-verbal responses including silences [23], which may signal reluctance, distress or fatigue. The author has over 20 years experience conducting research with people with disability including those with intellectual/cognitive impairments. She will ensure that interviews are tailored for each individual, taking into account their specific communication needs and level of understanding.

We will begin our life history interviews with a broad open-ended question “Tell me about…”. This unstructured start to each interview will provide participants with the opportunity to tell their story in their own words giving them an important degree of control over the interview. Further probing questions will explore how disability was/is viewed in each context with examples of past and present attitudes, practices, experiences and people who were/are supportive of and/or pose barriers to, participation and inclusion within each context. Participants will also be asked to reflect on what would have made their refugee journey smoother and less problematic. As recommended by Jessee [24], the final interview will conclude with a wrap up question encouraging participants to reflect on their life as a whole.

## 5. Data Analysis

The audio-recorded interviews in Arabic will be transcribed and then translated into English. The researcher and co-researcher will use qualitative software package NVivo12 to jointly analyse the English transcripts using coding and constant comparison techniques alongside the development of narratives [22,24]. This joint approach to coding increases rigor and trustworthiness of the analytic process [22]. Each of the participant’s three interviews will be coded and analysed individually and then together to develop the individual narrative. BenEzer and Zetter [13] advocate the creation of journey narratives as they enable “the individual to portray a multi-layered experience” (p. 313). Once this analysis is completed for each participant, constant comparison will be used to identify common and divergent themes related to participant journeys within and across the participant group [25]. This may result in a number of identifiable “disability refugee journey” narrative tropes. Jessee [24] describes this analytical approach as “a combination of reconstructive analysis, whereby the life history is used to reconstruct an approximation of participants’ lived experiences, and narrative analysis which ‘identifies and then explains the ways in which people create and use stories to interpret the world’ to create a ‘storied past’” (p. 437). A written version of each participant’s reconstructed story will be presented to them for verification and as a journey record. At the conclusion of the project, participants will also receive a de-identified summary of overall findings.

## 6. Discussion: Ethical Considerations and Dissemination

Ethical considerations are paramount in this study in which participants will likely describe traumatic and distressing events precipitating and resulting in their refugee journeys. Researchers therefore require great sensitivity. BenEzer and Zetter [13], also acknowledge the potential therapeutic benefits for participants in telling their journey narratives in a supportive environment. However, the particular circumstances of each individual’s refugee journey have the potential to make the person recognisable to others even with deidentification processes such as the use of pseudonyms. Crawley and Jones [14] also warn of the “importance of anonymity in the context of potentially clandestine journeys” (p. 3231). It is important that potential participants understand that while all measures to ensure anonymity will be taken, a potential for recognition still exists [23].

In general, there are a number of additional ethical considerations related to undertaking research that involves people with intellectual or cognitive disability, including, as already noted, explaining the research to participants and gaining informed consent, use of inclusive practices, safeguarding participants, protecting privacy and confidentiality and ensuring that participants benefit where possible from their involvement in the research [26]. However, it is also important to respect the right of people with intellectual and cognitive disability to participate in research, as documented in the CRPD.

The employment of a co-researcher who speaks Arabic and understands the cultural and historical contexts of participants is vital for ensuring people feel comfortable and are able to tell their experiences in their own language [20]. The Plain Language Information and Consent Statements approved by the author’s university Human Ethics Research Committee and provided in both Arabic and English have details about what is being asked of participants, their right to withdraw from the study at any time, and reassurance about anonymity in reporting. This detail is important to ensure that potential participants have enough information to make an informed decision about whether to participate or not [23]. As part of the inclusive approach, the consent and data collection processes are designed to offer repeated opportunities to explain the research, ascertain consent and provide information. At the beginning of each subsequent interview, the co-researcher will check that the participant still understands what they are participating in and is happy to proceed. All participants can elect to be supported by a trusted person of their choosing during provision of information, consent processes and/or interviews.

In keeping with the PAR approach, participants will be consulted about dissemination strategies. This may include the compilation of de-identified extracts of people’s life stories into one publication to be disseminated through refugee and disability organisations and online forums. There will also be peer-reviewed journal articles on which the co-researcher will be co-author. The outcomes will also be presented at refugee and disability conferences and other forums with the option of co-presentation depending on people’s preference and availability. If any part of the described protocol requires amendment during the study implementation, these changes will be documented and explained in future publication of results.

## 7. Conclusions

These life history interviews involving people with disability from refugee backgrounds have the potential to foreground the voices and experiences of people who are often absent, silenced or excluded in research and, in so doing, hopefully impact Australian refugee policy [11]. Soldatic et al. [11] noted that the current Australian five-year provision of resettlement services offered to new arrivals from refugee backgrounds is inadequate for many people with disability and their families due to the specific barriers they face. These barriers include the limited understanding on the part of disability services and policy makers about past experiences and current needs of this group. Research such as that referred to in this protocol paper will provide evidence to inform service providers and policy makers about the need to plan for and finance culturally appropriate, individually tailored, and long-term supports and services. This research also has the potential to highlight policy responses required for Australia to uphold its obligations for people with disability from refugee backgrounds under the CRPD [2] and the 1951 Refugee Convention [9].
